# Schizophrenia clinical symptom differences in women vs. men with and without a history of childhood physical abuse

**DOI:** 10.1186/s13034-016-0092-9

**Published:** 2016-03-03

**Authors:** Deanna L. Kelly, Laura M. Rowland, Kathleen M. Patchan, Kelli Sullivan, Amber Earl, Heather Raley, Fang Liu, Stephanie Feldman, Robert P. McMahon

**Affiliations:** Department of Psychiatry, Maryland Psychiatric Research Center, Spring Grove Hospital Grounds, University of Maryland School of Medicine, Box 21247, Baltimore, MD 21228 USA

**Keywords:** Abuse, Trauma, Schizophrenia, Women, Sex, Gender

## Abstract

**Background:**

Childhood abuse has been implicated as an environmental factor that increases the risk for developing schizophrenia. A recent large population-based case–control study found that abuse may be a risk factor for schizophrenia in women, but not men. Given the sex differences in onset and clinical course of schizophrenia, we hypothesized that childhood abuse may cause phenotypic differences in the disorder between men and women.

**Methods:**

We examined the prevalence of childhood physical abuse in a cohort of men and women with schizophrenia and schizoaffective disorder. Specifically, we examined differences in positive, negative, cognitive and depressive symptoms in men and women who reported a history of childhood physical abuse. We recruited 100 subjects for a single visit and assessed a history of childhood physical abuse using the childhood trauma questionnaire (CTQ) and clinical symptoms and cognition using the brief psychiatric rating scale (BPRS), the calgary depression scale (CDS) and the repeatable battery of the assessment of neuropsychological status (RBANS) for cognition.

**Results:**

Ninety-two subjects completed the full CTQ with abuse classified as definitely present, definitely absent or borderline. Twelve subjects who reported borderline abuse scores were excluded. Of the 80 subjects whose data was analyzed, 10 of 24 (41.6 %) women and 11 of 56 (19.6 %) men reported a history of childhood physical abuse (χ^2^ = 4.21, df = 1, p = 0.04). Women who reported such trauma had significantly more psychotic (sex by abuse interaction; F = 4.03, df = 1.76, p = 0.048) and depressive (F = 4.23, df = 1.76, p = 0.04) symptoms compared to women who did not have a trauma history and men, regardless of trauma history. There were no differences in negative or cognitive symptoms.

**Conclusions:**

Women with schizophrenia and schizoaffective disorder may represent a distinct phenotype or subgroup with distinct etiologies and may require different, individually tailored treatments.

## Background

Women and men with schizophrenia differ with respect to age at onset, disease course, clinical symptoms, cognitive and social performance, and neurobiological factors. Men diagnosed with schizophrenia typically present with an earlier age on onset, have poorer premorbid function, more negative symptoms, and lower social functioning [1, 2]. Comparatively, women are diagnosed at a later age, have more affective symptoms, and more social supports [3, 4]. An evaluation of 269 subjects (181 men, 88 women) registered with the Danish National Schizophrenia Project found that women were statistically more likely to be depressed, have delusions of guilt, unrealistic self reproach, and more accepting of their illness than their male counterparts both at baseline and 2 years after treatment [5]. However, a review of the literature indicated varying results in the clinical symptoms and course of illness in men in women, suggesting that schizophrenia is a heterogeneous disorder [6]. Sex differences have important consequences for understanding the etiology and course of schizophrenia, particularly since the incidence rate of early onset schizophrenia in women has been rising linearly since the 1970s, a phenomenon not seen in males [4].

Research has shown that schizophrenia and psychosis may be caused by different etiologic and pathophysiologic processing, including environmental determinants. Recent studies suggest that environmental exposures may play a more significant role in the underlying pathophysiology and/or etiology of schizophrenia than previously thought [7]. For example, Tienari et al. [8] found that the level of functioning in a nuclear family was more important than genetics. Specifically, offspring of parents with schizophrenia who had been raised by healthy adoptive families were less likely to be diagnosed with a psychotic disorder compared to those who had been maltreated by their adopted parents. Thus, abuse in childhood may increase vulnerability to schizophrenia, but evidence supporting a causal link has been inconsistent and data are limited by methodological concerns [9, 10]. One particular area that differs by sex is the occurrence of childhood physical and sexual abuse, which is consistently reported across geographic regions and cultures. Women with schizophrenia have higher rates of both types of abuse compared to men. Between 25–65 % of women with schizophrenia have reported a history of childhood abuse compared to 10–20 % of men [11–14].

Further, recent data are accumulating to suggest that exposure and sensitivity to stress may play a role in the etiology, neurobiology and course of schizophrenia [15]. Recently, the largest population-based case–control study of childhood abuse and psychosis to date found that schizophrenia is associated particularly with physical abuse in women but not men [12]. Additionally, rates of childhood abuse are significantly higher in women with schizophrenia compared to women in the general population, although rates of abuse among men with schizophrenia are similar to those among men in the general population [16]. After controlling for demographic variables, Briere et al. [17] found that physical and sexual abuse were the most powerful predictors of psychiatric symptoms in women with psychiatric disorders.

Others have reported on childhood abuse in schizophrenia but have not specifically examined differences by sex. Lysaker et al. [18] found that individuals with schizophrenia who had a childhood history of sexual abuse were less intimate interpersonal relationships. A later study by Lysaker et al. [19] found that individuals with schizophrenia who had been sexually abused in childhood had higher levels of dissociation, intrusive experiences, and anxiety than the non-abused schizophrenia group These clinical symptoms were different than those individuals with a history of childhood sexual abuse who had subsequently been diagnosed with post traumatic stress disorder (PTSD) and not psychosis. Chapleau et al. also found that schizophrenia patients with at least one traumatic experience had more alienation, insecurity and egocentricity than those without [20]. Further research has categorized the effect of certain types of trauma on quality of life measures, with research generally indicating poor quality of life and lower global assessment of functioning [21]. For example, individuals with schizophrenia who reported sexual trauma had overall poorer levels of general health, mental health, and role function whereas those who had reported trauma related to harm to others reported poor general health, social function, emotional- and physical-related role functions [22].

The main objectives of this study were to examine the rates of childhood physical abuse and clinical symptom profiles in both women and men with schizophrenia. Physical abuse is the most consistently and commonly reported type of abuse in most population samples and hypothesized to be reported equally in men and women [23]. We hypothesized that women with schizophrenia who have a history of childhood abuse have a distinct phenotype not seen in men. This may have practical implications in the success of treatments available for this population.

## Methods

One hundred individuals with a DSM-IV diagnosis of schizophrenia or schizoaffective disorder were recruited from the inpatient and outpatient setting. Individuals were greater Baltimore area through clinics affiliated with the Maryland Psychiatric Research Center. Subjects were between 18–75 years old and able to provide informed consent. During the consent process, participants were made aware that they would be asked questions about childhood abuse. However, they were not recruited on the basis of their history of abuse, so as to avoid biasing recruitment towards over or under representation of childhood abuse. The protocol was approved by the University of Maryland Institutional Review Board and all research subjects signed informed consent prior to participation in the study.

Childhood abuse history was evaluated using the childhood trauma questionnaire (CTQ). The CTQ includes specific objective items that rate the history of childhood abuse across five domains, namely emotional abuse, physical abuse, sexual abuse, emotional neglect, and physical neglect. There are five questions for each domain and three items of minimization or denial to detect false negative reports. Each of these 28 items was rated by participants on a five point Likert scale with response options of never true, rarely true, sometimes true, often true and very often true. Choice of a high cut off for definition of abuse (only regular, frequent or very frequent) enhances the validity of the exposure assessment [24].

The CTQ is a validated measure that assesses trauma and abuse and has been validated across diverse populations. Bernstein et al. examined the validity of the CTQ across four diverse populations and found that each group responded to the scale’s items in a reasonably equivalent manner and demonstrated good criterion-related validity [25]. Further, it has been reported by Bendall et al. as the measure of choice in assessing childhood abuse in individuals with psychotic disorders [10]. Self-reporting of abuse by people with schizophrenia has been shown to be reliable over time, and as reliable as reports collected from the general population [26, 27]. The validity of the CTQ in schizophrenia has been shown and the credibility of the methodology has been established [11, 12, 28]. In several studies, 75 % of psychiatric patient self-reports match their objective abuse history [29–32].

Our study focused exclusively on the physical abuse domain as determined by scoring criteria [33]. Physical abuse has been found to have the highest predictive value to schizophrenia [12]. We included a score of ≥10 (moderate to severe) to indicate a history of childhood physical abuse and a score of ≤7 as having no such abuse. To ensure that we did not include individuals with a tendency to give socially desirable responses or individuals likely to produce false-negative reports, we examined the three items that contained the minimization/denial scale. Individuals who endorsed one on this scale were excluded, as they were likely to give exaggerated or desirable responses rather than actual item content. This characterization has been described in the manual [33].

In addition to the CTQ, the subjects also completed the brief psychiatric rating scale (BPRS) [34], the calgary depression scale [35] (CDS) to assess psychotic and depression symptoms and the repeatable battery of the assessment of neuropsychological status (RBANS) to assess cognitive function [36]. Raters for the BPRS were trained and reliable with intraclass correlation coefficients (ICC) of >0.8 compared to a consensus of experienced raters.

For statistical analysis, we fitted a two-way analysis of variance (ANOVA) model comparing differences in demographic variables, clinical symptoms, and cognitive scores in women and men, who did or did not report childhood physically abuse. In instances where abuse history by sex interactions were present, we performed post hoc t-tests comparing scores of reported abuse history separately in women and men. Those with scores of possible or borderline abuse were excluded.

## Results

Of the 100 subjects recruited, 92 completed the full CTQ. Of these, 12 participants (six women and six men) were excluded, as their abuse history was questionable. A total of 80 participants were included in the analysis. Ten of 24 (41.6 %) women and 11 of 56 (19.6 %) men reported a history of physical abuse (χ^2^ = 4.21, df = 1, p = 0.04). Results were assessed by sex and abuse history, leading to four separate categories: women who had been physically abused, women who had not been physically abused, men who had been physically abused, and men who had not been physically abused. There were no statistical differences between the four groups in age, race, and level of education (see Table 1). None of the patients had a documented diagnosis of post traumatic stress disorder (PTSD).

**Table 1:** Demographic and clinical variables by abuse group

Variable	Women physically abused	Women not physically abused	Men physically abused	Men not physically abused	Statistical tests
	N = 10	N = 14	N = 11	N = 45	
Age (years)	37.8 ± 10.8	32.6 ± 11.9	30.9 ± 7.7	31.6 ± 9.8	Women: t = −1.25, p = 0.22 Men: t = 0.23, p = 0.82 Abuse x sex interaction: F = 1.24, df = 1,76, p = 0.27
Race	White (50.0 %)	White (64.3 %)	White (63.6 %)	White (62.2 %)	Chi square t = 0.29, df = 2, p = 0.87
Level of education (years)	12.7 ± 2.5	11.7 ± 2.9	11.8 ± 1.3	12.3 ± 1.8	Women: t = −1.17, p = 0.24 Men: t = 0.75, p = 0.46 Abuse x sex interaction: F = 1.92, df = 1,75, p = 0.17
BPRS total score	34.7 ± 8.6	30.2 ± 4.0	30.6 ± 8.4	31.2 ± 5.8	Women: t = 1.71, p = 0.09 Men: t = −0.26, p = 0.79 Abuse x sex interaction: F = 2.24, df = 1,76, p = 0.14
BPRS psychotic symptom	9.7 ± 4.6*	6.9 ± 2.9	7.2 ± 3.3	8.1 ± 3.3	Women: t = 1.96, p = 0.05 Men: t = −0.77, p = 0.443 Abuse x sex interaction: F = 4.03, df = 1,76, p = 0.048
BPRS negative symptom	5.7 ± 2.4	6.3 ± 2.8	7.5 ± 2.6	6.9 ± 2.5	Women: t = −0.56, p = 0.58 Men: t = 0.61, p = 0.54 Abuse x sex interaction: F = 0.67, df = 1,76, p = 0.42
CDS	3.3 ± 2.8*	1.4 ± 1.5	1.3 ± 1.4	1.6 ± 2.1	Women: t = 2.28, p = 0.03 Men: t = −0.45, p = 0.652 Abuse x sex interaction: F = 4.23, df = 1,76, p = 0.04
RBANS total	70.1 ± 13.7	70.4 ± 13.2	75.2 ± 19.6	74.1 ± 16.1	Women: t = 0.04, p = 0.971 Men: t = −0.19, p = 0.847 Abuse x sex interaction: F = 0.02, df = 1,71, p = 0.88

* Signficantly greater value as determined by the abuse x sex interaction

### Psychiatric symptoms

Women with a history of physical abuse had comparable total BPRS scores and negative symptoms compared to women without a history of physical abuse and men, regardless of physical abuse history (see Table 1). However, women with a history of childhood physical abuse had significantly more positive psychotic (F = 4.03, df = 1.76, p = 0.048) and depressive (F = 4.23, df = 1.76, p = 0.04) symptoms compared to the other three groups. Specifically, mean BPRS psychotic symptom scores were 9.7 ± 4.6 in females with physical abuse histories versus mean scores between 6.9 and 8.1 in the three other groups. Mean scores on depressive symptoms, as measured by the CDS, were 3.3 ± 2.8 in women with abuse, more than twice as high as any of the other three groups. Lastly, the RBANS total score did not have a sex by abuse history interaction (F = 0.02, df = 1.71, p = 0.88). There were also no differences among the domains of the RBANS.

## Conclusions

Women with schizophrenia were more likely to report a history of childhood physical abuse compared to men with schizophrenia. Moreover, these women experienced significantly more positive psychotic and depressive symptoms compared to women without a history of abuse and men, regardless of abuse history. Women with schizophrenia and a history of childhood abuse may represent a distinct subgroup. Understanding the pathophysiology and clinical symptoms in these women may facilitate the development of tailored treatments.

Women may have an underlying biological or genetic mechanism that predisposes them to schizophrenia when coupled with physical abuse. This was suggested by Fisher et al. [12] in the *British Journal of Psychiatry,* which suggested an increased risk of schizophrenia in present women with childhood abuse histories but not men. Adults with schizophrenia and a history of childhood physical or sexual abuse may experience more psychotic symptoms—specifically auditory hallucinations—than adults without abuse histories [27, 37–41]. Women with schizophrenia have reported higher rates of auditory hallucinations, persecutory delusions, and less negative symptomatology compared to men [42–45]. However, potential sex differences in symptoms and childhood abuse has not been well-documented. Those studies that document abuse history in individuals with schizophrenia do not distinguish by sex.

Given the differences in clinical symptoms by sex and abuse history, women who have been abused may present a potentially distinct phenotype, for which tailored treatments may be developed. As such, this potential distinct phenotype deserves in depth examination to better understand the role of physical abuse on childhood and adolescence neurodevelopment, and the subsequent presentation of schizophrenia.

One hypothesis by which a history of childhood abuse can increase the risk of schizophrenia in women may be through hormones and a diathesis stress model. Developmentally, gonadal hormones may be a mechanism through which the observed sex differences in psychotic symptoms can occur. The brain undergoes significant and sex-specific, development during maturation and early childhood. Abuse could play a role in later reactivity to stress through the interplay of gonadal hormone differences. The adolescent period is a critical period in which the gonadotropin-releasing hormone (GRH) secretion from the hypothalamus triggers a host of hormone dependent processes. During adolescence, the hypothalamic–pituitary–adrenal axis (HPA) function undergoes prolonged activation in response to stressors, which differs from adults, and may play a role in the ongoing development of brain. Romeo et al. [46, 47] found that, in response to an acute stressor, glucocorticoid release is prolonged in juvenile rats compared to adult rats. In a clinical study, the severity of a woman’s abuse was positively correlated with baseline stress hormones, such as corticotropin releasing hormone, while these same factors were *not* positively correlated in men [48].

Similar models have been developed in previous research. Read, Perry, Moskowitz, and Connolly hypothesized the “traumagenic neurodevelopmental model” in which early traumatic events precede the onset of schizophrenia. Increasing levels of stress can lead to over-reactivity of the HPA axis, leading to structural brain changes and abnormalities in the neurotransmitter systems. Specifically, stress can lead to increased release of adrenocortropic hormone (ACTH) from the pituitary and release of glucocorticoids from the adrenal cortex [49]. This leads to excessive release of cortisol, dopamine. With repeated, excessive stress, the negative feedback system that dampens the HPA axis is impaired. Other studies have shown that abused girls have greater synthesis and higher levels of dopamine, norepinephrine, and epinephrine. Further, the hippocampus which is very sensitive to stress activation, can be permanently reduced. The release of stress hormones can lead to physiological changes. For example, Rajkumar found that physical abuse was linked to elevated systolic blood pressure in women [50].

Subdividing schizophrenia into distinct subtypes can reduce inter-individual variability or heterogeneity, which may suggest differential etiologies. A recent small study (N = 28) also found that childhood abuse predicted more positive and depressive symptoms as well as reduced whole brain volumes, a reduction that correlated with cortisol levels in people with schizophrenia [51]. We are following up these initial findings by studying a stress paradigm in women and men with and without childhood abuse to assess differences among groups to physiological response to psychological stress. We hope to elucidate mechanisms that could lead to different illness presentation and individualized pharmacological and psychosocial interventions to minimize the effects of abuse on mental illness burden. We note that this phenomenon of positive and depressive symptoms seen may not be specific to schizophrenia. These types of symptoms are seen in PTSD and depressive patients [52–55], thus, the pathophysiology may cut diagnostic boundaries, such that childhood abuse may broadly effect circuitry and brain development that could lead to a variety of psychiatric illness disturbances. Thus, broader attention beyond its relationship to schizophrenia is crucial that would allow for subgroup classification that may be in the national institutes of mental health research domain criteria (RDoC) framework.

This study contributes to the growing research on the effect of childhood abuse on clinical symptoms in men and women with schizophrenia. However, it is not without limitations. First, the number of study subjects was relatively small. Although 100 subjects were recruited for the study, 92 completed the study and 21 reported a history of childhood physical abuse. Second, the study did not examine other types of abuse and its potential impact on psychotic and affective symptoms. Third, the study used the CTQ to assess trauma history among subjects. While the CTQ is well-validated and has proven to be a good measure to assess trauma in individuals with schizophrenia, it does not assess the severity of trauma as it relates to the severity of psychotic symptoms. Also, we have used the BPRS to measure positive symptoms as our raters are experienced, trained and reliable on this measure. Other rating scales may be more comprehensive to capture positive symptoms such as the scale of the assessment of positive symptoms (SAPS) or positive and negative symptom scale (PANSS). Likewise the findings may go beyond positive and depressive symptoms and we did not measure variables such as object relations deficits or quality of life. Finally, although none of the subjects had a documented PTSD diagnosis, a structured clinical interview for DSM disorders (SCID) was not performed to exclude this diagnosis formally.
